# The Board of Regents and Medical Colleges

**Published:** 1882-03

**Authors:** 


### THE BOARD OF REGENTS AND MEDICAL COLLEGES.

The following act has been introduced into both Houses of the Legislature:

SEC. 1. The Regents of the University of the State of New York may authorize duly incorporated colleges and universities of this State to establish and maintain medical departments at the places where such colleges or universities are located by law, or at places to be designated by said Regents other than those wherein such colleges or universities are so located, and they may authorize such colleges or universities to maintain other departments at places to be designated by said Regents.

SEC. 2. This act shall take effect immediately, and all acts and parts of acts inconsistent with this act are hereby repealed.

The real object of this bill has been clearly made known by Assemblyman Hickman, of this city, in quite a lengthy interview published in the Buffalo *Daily Express* of February 20th. The courts having interposed their authority in the matter of the College of Physicians and Surgeons of this city, whereby it has been found impracticable to continue under existing laws either independently or as a department of a duly incorporated university, it is designed by interested parties to change the statute in order that this institution, now moribund, may be revived under fresh auspices or a new medical college organized and connected with Alfred University as its medical department. It having been claimed that the Regents requested this change in the law, we communicated with the Secretary, Dr. Murray, upon this subject, and have received the following in reply:

*Editors Buffalo Medical and Surgical Journal:*

GENTS—In reply to yours of the 20th inst., I desire to say at once that the question as to allowing universities to establish medical departments at places other than the seat of the university, has never been considered by the Board of Regents, and there has been no occasion to hold or form an opinion thereon. No one has a right to say that they desire a change in the statute relating to that subject. If the Legislature, or a committee of the Legislature, asks for an opinion from the Board on that subject. doubtless they will be ready to give it, as it is a matter of great interest to the body of institutions connected with the Board of Regents. But of the measure introduced into the Legislature, to which your enquiries refer, they have no knowledge, and certainly no responsibility for it.

Very respectfully your obedient servant,

DAVID MURRAY, Sec'y.

Further comment upon this subject at this time is unnecessary. The motives actuating the originators of this measure are too plain to be successfully hidden by the transparent explanations made by Mr. Hickman, the only member of the lower house who is especially anxious for its enactment. The profession, without distinction, are unanimous in opposing this law. We trust that wise counsels will prevail in matters which concern the vital interests of medical education in this State.

